# Assessment of Techno-Functional and Nutraceutical Potential of Tomato (*Solanum lycopersicum*) Seed Meal

**DOI:** 10.3390/molecules25184235

**Published:** 2020-09-15

**Authors:** Ramón Maldonado-Torres, Jocksan I. Morales-Camacho, Fernando López-Valdez, Luis Huerta-González, Silvia Luna-Suárez

**Affiliations:** 1 Centro de Investigación en Biotecnología Aplicada, Instituto Politécnico Nacional, CIBA-IPN, Tepetitla, Tlaxcala 90700, Mexico; lic.rmaldonadot@gmail.com (R.M.-T.); flopez2072@yahoo.com (F.L.-V.); lhuertaglez@gmail.com (L.H.-G.); 2 Departamento de Ingeniería Química, Alimentos y Ambiental, Universidad de las Américas Puebla, Sta. Catarina Mártir, San Andrés Cholula, Puebla 72810, Mexico; jocksan.morales@udlap.mx

**Keywords:** tomato seed meal, techno-functional properties, nutraceutical properties, antihypertensive activity, *Lactobacillus* sp.

## Abstract

Tomato (*Solanum lycopersicum*) is a widely consumed fruit all around the world. The industrial exploitation of tomato generates a lot of waste. Most of the utilization of tomato seeds waste is focused on animal feeding, as well as a food ingredient aimed to increase the protein content, and raw material for some organic bioactive component extraction. The aim of this work was to evaluate the techno-functional properties of tomato seed meal (TSM) and its nutraceutical properties after applying defatting processing (TSMD), and to evaluate the nutraceutical properties after a fermentation processing (TSMDF) by *Lactobacillus* sp. The results showed that, at alkaline conditions (pH 8–9), the techno-functional properties for TSM and TSMD improved. In comparison with TSM, TSMD showed higher water holding capacity (WHC ≈32%), higher oil holding capacity (OHC ≈13%), higher protein solubility (49–58%), more than 10 times foaming activity (FA), more than 50 times foam stability (Fst), as well as an improved emulsifying activity (EA) and emulsion stability (Est) wich were better at pH 9. Regarding the nutraceutical properties, after 48 h of fermentation (TSMDF), the antioxidant activity was doubled and a significant increase in the iron chelating activity was also observed. During the same fermentation time, the highest angiotensin-converting enzyme inhibition (ACEI) was achieved (IC50 73.6 μg/mL), more than 10 times higher than TSMD, which leads to suggest that this fermented medium may be a powerful antihypertensive. Therefore, the strategy proposed in this study could be an option for the exploitation of tomato wastes.

## 1. Introduction

Tomato (*Solanum lypersicum*) is considered as a fruit of high consumption and production all over the world. It is also known that its center of origin comes from the American continent, between Peru and Mexico [1]. In 2018, annual world production of tomato was reported to be around 182,256,458 tons. The main producer countries from America are USA, Mexico, and Brazil with 6.9, 2.5 and 2.25%, respectively, from total world production [2]. The production is mainly aimed to produce sauces, pastes, juices, puree, and others which generates 3–5% residues (mainly seed and peel) from raw material [3]. Therefore, the main producer countries from America could be generating between 638,452 and 1,064,072 tons of residue annually, of which tomato seeds represent 60% [4]. Seeds from tomato are rich in components which have nutritional, techno-functional, and some nutraceutical properties that have been highlighted in recent studies [5,6]. Thus, in order to reduce environmental problems by tomato wastes and considering properties from tomato seeds, processors and researches have focused on the use of tomato seeds for animal feeding, as an ingredient for bakery products, and as a raw material for the extraction of oil and proteins [7].

Most alternative uses of seed wastes from tomato are focused on animal feeding due its high protein level content, which is used as a supplement for animal feeding because it improves the production and fat content of cow milk and the growth of sheep [8,9]. Some studies have used tomato pomace meal and tomato seed powder to formulate crackers and bread, respectively. The replacement of wheat flour increased the protein content in new products, but acceptability may be compromised to some extent by changes in the texture and flavor quality of new products [10,11].

The techno-functional properties of proteins from food can be classified according to the interaction they establish with the medium [12]. Hydration properties refer to the protein’s ability to interact with water by forming hydrogen bonds. Protein–protein interactions refer to the ability to form bonds or to bind to other proteins present in the medium, either by hydrophilic or hydrophobic interaction. Surface properties refer to the ability to keep two immiscible phases together by directing the fluids towards the interface, resulting in a new structure known as emulsion [13]. Some studies have reported that protein concentrate extracted from tomato seed showed oil absorption capacity, foaming capacity, emulsifying capacity, and stability, although these techno-functional properties were influenced by extraction conditions as reported as temperature, pH, chemical composition of solvent for extraction, and others [7,14]. Therefore, the conditions processing for tomato seed meal can affect the techno-functional properties.

The nutraceutical properties of a food are related to the specific composition. Different studies have been reported that the phenolic content from peels of tomato pomace have antioxidant activity by radical scavenging inhibition. Other studies reported that carotenoids from tomato promote health benefits to reduce risks of several diseases, such as Alzheimer’s, cardiovascular diseases, and cancer, mainly by lycopene from pulp and peel [7]. With regard to proteins, it refers to the ability of bioactive peptides that are obtained by biochemical fermentation or by enzymatic hydrolysis, and contributes to some physiological benefits as antidiabetes, antiinflammatory, antihypertensive, antioxidative, among others. These properties are related with their structure and its peptide sequence which can act to block the formation of the radical from precursors, such as superoxide or inhibiting angiotensin converting enzyme (for antioxidative effect and antihypertensive affect, respectively) [15]. Recently, some works have identified some antioxidant peptides from tomato isolate from seeds, and the results showed potential scavenging capacity by peptides [16]. A way to obtain bioactive peptides is during fermentation by lactic acid bacteria (as *Lactobacillus* spp.) which produce proteolytic enzymes and hydrolyze proteins that generate peptides or amino acids with bioactive properties for health benefits [17].

The aim of this work was to evaluate the techno-functional properties of tomato seed meal (TSM) and its nutraceutical properties after applying defatting processing (TSMD) and to evaluate the nutraceutical properties after a fermentation processing (TSMDF) by *Lactobacillus* spp. For this, techno-functional properties, such as water/oil capacity, protein solubility, foaming capacity, and emulsifying capacity, were measured for TSM and TSMD. Nutraceutical properties, such as DPPH radical scavenging, ABTS radical scavenging, Iron chelating activity, and angiotensin converting enzyme inhibition (ACEI), were determined for TSM, TSMD, and TSMDF.

## 2. Results

### 2.1. Sample Preparation and TSM Composition

The wet method used in this study showed a seed separation efficiency 54.05%. Proximal analysis of TSM showed that protein (26.93 ± 0.12%) and fiber (26.99 ± 2.92%) are majority components into seed meal (Table 1). The phenolic compounds into TSM was 53.38 mg Eq. GA/100g sample.

**Figure 1:**
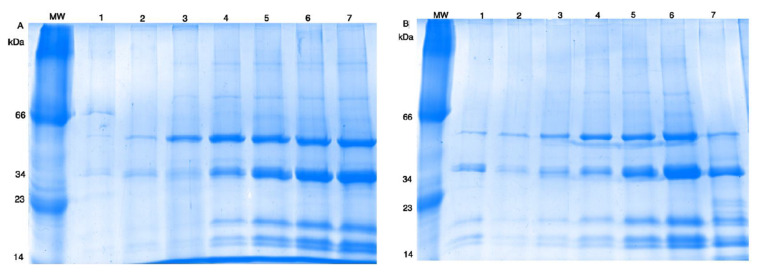
SDS-PAGE, protein solubility at different pH values. (**A**) TSM sample; (**B**) TSMD sample. Lanes: MW, molecular weight; 1, pH 3; 2, pH 4.5; 3, pH 5; 4, pH 6; 5, pH 7; 6, pH 8; 7, pH 9.

Electrophoretic pattern by SDS-PAGE gels showed a higher protein content from TSM and TSMD at pH from 6–9. As shown in Figure 1, five majority protein bands in both samples (TSM or TSMD) were around 16, 18, 22, 40, and 52 kDa were observed.

### 2.2. Techno-Functional Properties

#### 2.2.1. Water/Oil Holding Capacity: WHC and OHC

WHC showed significant differences between TSM and TSMD samples (*p* < 0.05). TSM samples did not show significant differences from pH 3 to 9 (3.18–3.27 g water/g sample). Likewise, TSMD samples showed a higher WHC (4.2–4.3 g water/g sample) from pH 6 to 9, at lower pH WHC slightly lower, but also showed significant differences compared to TSM samples (Supplementary materials Figure S1A). Table 2 shows that, in both samples, WHC is higher as pH increases.

OHC was higher in TSMD samples at the pH value tested (5, 5.5, and 6), and the results ranged from 2.26 to 2.5 g oil/g sample, while OHC in TSM ranged from 2 to 2.2 g oil/g sample. Significant differences (*p* < 0.05) between TSM and TSMD were observed at pH 5.5 and 6 (Table 2 and supplementary Figure S1B).

#### 2.2.2. Protein Solubility

Protein solubility increased with pH. In both samples, at pH values from 3 to 5, protein solubility was lower than 50% (Supplementary Figure S2). Maximum protein solubility was reached in TSMD samples at pH 8 and 9 with 79.4 and 83.7%, respectively. At the same pH values, TSM samples showed a protein solubility between 50.1 (pH 8) and 55.9% (pH 9) (Table 2). These observations were confirmed with SDS-PAGE gels, which showed greater intensity in protein bands as pH increased, that is at a pH greater than 6 (Figure 1).

#### 2.2.3. Foaming Capacity

No foaming activity (FA) was found in TSM samples. Instead, TSMD showed FA as from pH 5 to 9. A maximum FA was reached at alkaline conditions whereby, at pH 8, it was 26.3% and at pH 9, it was 32.1% (Table 2). These results are coincident with foam stability (Fst), which was higher at higher pH. Foam half-time at pH 8 was 29.3 min and at pH 9 was 38.3 min. As can be seen, the foaming capacity of the TSMD sample was higher at alkaline conditions.

#### 2.2.4. Emulsifying Capacity

**Figure 2:**
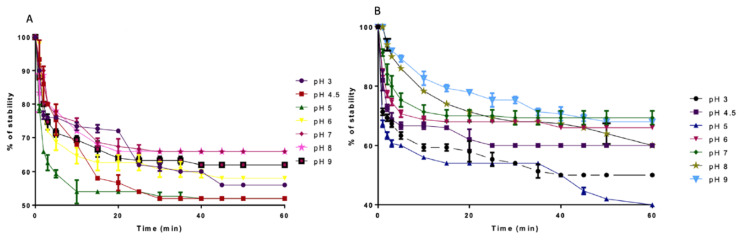
Emulsifying stability at different pH values. (**A**) TSM sample; (**B**) TSMD sample.

Table 2 shows the results obtained with respect to emulsifying capacity. As can be seen, emulsifying activity (EA) in TSM starting from pH 7 (20%) was increasing at pH 8, showing an EA value of 87.5%, whereas at pH 9, the EA value decreased at 33.3%. Emulsion stability (Est) of TSM samples was around 68% at pH 7–8, and in a similar way to EA, the Est value was lower (62%) at pH 9. Similar results were observed for TSMD samples whereby, at pH 8, a maximum EA value with 87.5% was reached, and also at pH 9, a lower EA value with 60.5% was observed (Table 2). TSMD samples showed an Est value from 60.1 to 71.7%, and at pH 7 the maximum Est was value reached. For emulsions prepared using TSM, a reduction stability ≥30% during the first 10 min was observed (Figure 2A). However, for TSMD samples the reduction was lower than 30% in the same lapse time (Figure 2B). In both TSM or TSMD, at pH lower than 7, emulsifying activity was affected (Supplementary Figure S3).

Mean of triplicate assays ± standard deviation. Values with different letters within rows indicate significant difference (*p* < 0.05)

### 2.3. Nutraceutical Properties

The antioxidant capacity of TSM determined by the DPPH and ABTS methods showed an inhibition percentage of 41.5% (375.6 μmol Eq Trolox/100 g sample) and 20.2% (187.8 μmol Eq Trolox/100 g sample), respectively. Chelating activity observed for TSM sample was very small 1.1 % (Table 3). TSMD showed slightly lower scavenging activity than TSM for DPPH and ABTS reaching 38.9 and 19.3%, respectively. Iron chelating activity showed 2.2 and, 7.9% of angiotensin converting enzyme inhibition (ACEI).

### 2.4. Enhancement of Nutraceutical Properties by Fermentation

**Figure 3:**
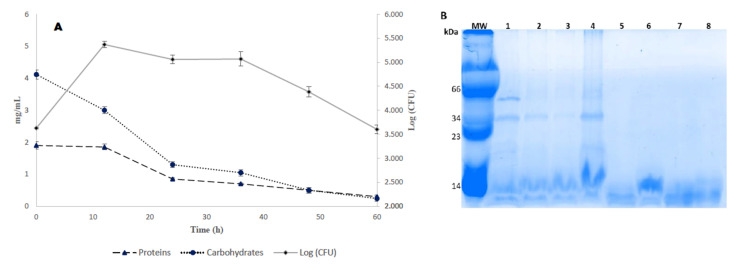
Kinetic of fermentation by *Lactobacillus* sp. (**A**) *Lactobacillus* sp growth and consumption of proteins and carbohydrates. (**B**) SDS-PAGE protein profile along fermentation process. Lanes: MW, molecular weight; 1, TSMD before sterilization; 2, TSMD sterilized; 3, 0 h fermentation; 4, 12 h fermentation; 5, 24 h fermentation; 6, 36 h fermentation; 7, 48 h fermentation; 8, 60 h fermentation.

Fermentation kinetic showed that *Lactobacillus* sp. achieved a maximum growth at 12 h after fermentation started, and the growth was stationary until 36 h (Figure 3A). In addition, the content of protein and carbohydrates drastically dropped between 12 and 24 h of fermentation, where the reduction was around 50% with respect to initial content (Figure 3A). Also, SDS-PAGE gel shows changes in the molecular weight distribution of proteins throughout the fermentation process, which could be the result of *Lactobacillus* sp. metabolism (Figure 3B).

After sampling, TSMDF showed better nutraceutical properties after 36 h of fermentation. The maximum values were registered at 48 h. DPPH and ABTS scavenging activity was almost double that shown by TSM and TSMD. Iron chelating activity and ACEI were 41.2 and 83.7%, respectively. As can be seen, all nutraceutical properties tested were improved during *Lactobacillus* sp. fermentation (Table 3).

DPPH radical scavenging; ABTS radical scavenging; ACEI, Angiotensin Converting Enzyme inhibition.

All assays were done using 0.01 mg/mL concentration protein based on the samples.

Mean of triplicate assays ± standard deviation. Values with different letters within columns indicate significant difference (*p* < 0.05)

### 2.5. In Silico Analysis Biopeptides

**Figure 4:**
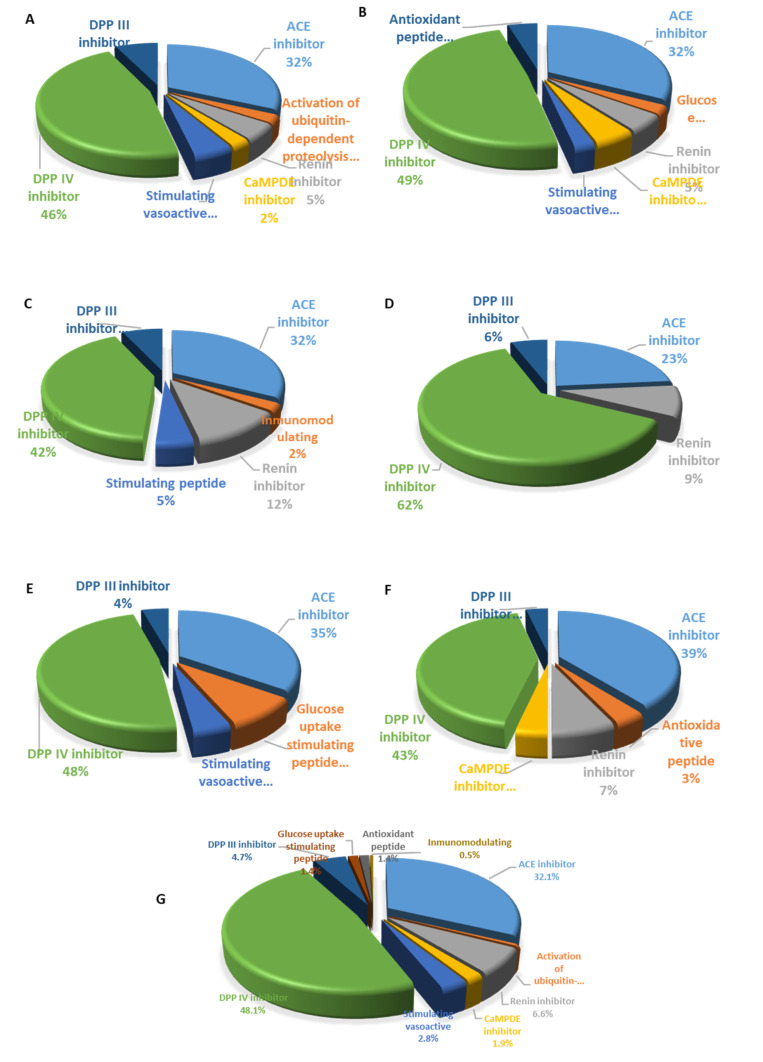
Frequency activity of biopeptides obtained by in silico proteolysis. (**A**) A5 protein; (**B**) W4 protein; (**C**) W15 protein; (**D**) S6 protein; (**E**) 4I8Q protein; (**F**) 6L4M protein; (**G**) Total frequency activity of peptides generated by hydrolysis in silico analysis using the six proteins from tomato seed.

In silico analysis showed that the TSM sample could be a potential precursor of antihypertensive peptides through the fermentation process. ACE inhibitor peptides showed a frequency activity ranged from 23 to 39%, being the second most predominant nutraceutical property (Figure 4), and in an interesting way DPP-IV inhibitor was the main nutraceutical property suggested by in silico evaluation (Figure 4G). In the proteolysis of the seed storage proteins from tomato, the frequency activity of ACE inhibitor peptides was 32% (Figure 4A–C), while a higher ACE inhibitor peptides frequency was obtained for vicilin (PDB6L4M) and aminoaldehyde dehydrogenase (PDB4I8Q) proteins from tomato seed (Figure 4E,F).

## 3. Discussion

### 3.1. Techno-Functional Properties

The separation method used to obtain peel and seeds is relevant to establish the steps of a methodology for the use of tomato wastes. In this sense, the value of seed separation efficiency reported in this study (54.05%) is higher than 22.4–24.2% and 51.99% reported by Shao et al. [18], and higher also than the seed separation efficiency (48.29%) reported by Kaur et al. [19], who used a mixing tank impeller. It has been reported that wet separation is recommended to acquire seeds, but production of sewage could be an environmental burden [7]. Then, an option could be to recover and recirculate the water during the separation process, as was done in this study.

Techno-functional properties are very important characteristics to maintain the desired texture and flavor of food products. In this sense, WHC and OHC are important to maintain or improve softness, mouthfeel, sensory acceptability, and others. Some studies have reported that defatted tomato seed of tomato pomace has better water absorption capacity, i.e., 3.35–3.45 (g water/g flour), than WHC for soybean protein isolate with 2.39 g water/g isolate, and even whole tomato seed of tomato pomace showed higher values for WHC (2.6 g water/g flour). In this study, results obtained for TSM and TSMD are around 22–25% higher for water holding capacity than those reported by Shao et al. for whole and defatted conditions, respectively [14]. Kang et al. [20] determined a water absorption of 2.06 g water/g flour for defatted soybean flour. Then, TSMD showed two times higher water absorption (4.2 g water/g flour). According to the results obtained, both TSM and TSMD samples could be used as a thickener for food viscous products as sauces or soups, which require WHC ranging from 1.97 to 4.72 g water/g sample [21].

Higher WHC values in TSMD sample can be explained by the fact that, in this sample, there is a greater content of proteins and their hydrophilic amino acids could be exposed at a pH higher than 5. Moreover, carbohydrates have hydrophilic regions as polar and charged side chains which can bind to more water molecules [14,22].

OHC is another important property exploited to formulate sausages, salad dressings, as a meat extender, for flavor retention, and for the enhancement of mouthfeel [23]. As seen in Table 2, TSMD sample showed similar result (2.36 g oil/ g sample) as reported by Shao et al. [14]. On the other hand, the results for TSM and TSMD are higher than those reported for soybean flour at 1.36 g oil/ g sample. Some studies have reported that OHC values increase proportionately with the protein content [24]. So, the higher OHC values observed for TSMD could be the result of the defatted process, because reducing the fat proportion increases the protein proportion, which allows non-polar amino acids or hydrophobic domains to be exposed and establish hydrophobic interactions, binding more oil molecules [23].

Protein solubility is important for its techno-functional applications in different foods. As can be seen, TSM and TSMD showed a high solubility at alkaline pH values (8–9), where the protein solubility is pH dependent. It is possible that the isoelectric point of proteins into tomato seeds is around pH 3–5. Therefore, by increasing the pH value, the net charge of the protein is increased as well as the protein solubility and flexibility, resulting in a higher foam capacity at pH ranging from 6 to 9. In addition, it is known that foam formation requires protein solubilization in the aqueous phase to form a layer of protein that stabilize the air droplets, which was observed in TSMD at greater pH values [21]. Foam stability (Fst) shown by the TSMD sample is similar to that reported for proteins isolated from defatted of tomato seeds, which showed better foam stability at alkaline conditions (pH 9–11) with lower foam stability than TSMD, ranging from 22 to 28 min as the half-life of foam [14]. Some natural oils and its component as fatty acids have been reported to function as antifoam agents [25]. In this sense, the TSM sample did not show foam capacity, maybe due its oil component.

The protein solubility reported by Elsohaimy et al. [26] for quinoa isolate showed a maximum solubility at pH 10. Other studies reported that the protein solubility shown by amaranth, buckwheat, and quinoa ranged from 2 to 35% in pH 3 to 5, in contrast with alkaline conditions, in which the protein solubility ranged from 50 to 100% [27]. In all these sources, the main protein fraction is composed of globulins assembled as a hexamer or trimer, which have been reported as storage proteins in seeds. Each hexamer subunit consists of an acidic (≈32–35 kDa) and basic subunit (≈20–24 kDa) linking by a disulfide bridge. Trimer globulins, e.g., from soy, are conformed by α, α’, and β, with a molecular weight of 72, 68, and 52 kDa, respectively [28,29]. These observations are according with the electrophoretic pattern observed in proteins of TSM and TSMD samples, so the majority of proteins observed maybe storage proteins from tomato seeds (Figure 1).

Emulsions mediated by proteins are weak or null at their isoelectric point because, in this condition, protein cannot diffuse to the oil–water interface [30]. This could be the reason why TSM and TSMD showed lower emulsifying activity (EA) at pH 3–5 and a higher EA value as the pH increased (Table 2 and Supplementary Figure S3).

The results obtained were partially in agreement with Shao et al. [14] who reported an EA at pH 9 of around 40 and 73% for tomato seed of tomato pomace and defatted tomato seed of tomato pomace, respectively. While for TSM and TSMD the maximum EA was achieved at pH 8 with 87.5 and 83.3% respectively, both samples showed an emulsifying stability (Est) at pH 7, which is similar to that reported by same authors. Like foaming capacity, EA of TSM and TSMD is pH-dependent because, as pH increases, EA was increased, and alkaline conditions improved this property in both samples. This may occur because emulsion activity is related to hydrophilic-hydrophobic balance of proteins, the orientation of hydrophilic (to aqueous phase) and lipophilic (to oil phase) amino acids, and the protein solubility which are affected by pH [23]. Therefore, it is possible that, at pH 8, as proteins from TSM and TSMD showed greater solubility and find conditions to maintain hydrophilic–hydrophobic balance, proteins adopt a structure conformation that allowed for higher emulsifying activity. The EA value for TSM was higher than for TSMD, probably due to the difference in constituents, such as oil or fat, that can also affect emulsifying properties [27].

### 3.2. Nutraceutical Properties

Antioxidant capacity and scavenging activity could be the result of a protein fraction present in TSM and TSMD according to Mechmeche et al. [14], who reported a protein isolate from tomato seed. Valdez-Morales et al. [31] reported an antioxidant capacity in tomato seeds var. saladette by DPPH (92.4 μμM Eq Trolox/100 g sample) and ABTS (67.4 μμM Eq Trolox/100 g sample) methods, which are almost three and four times lower than the results obtained for TSM and TSMD in this report. The higher DPPH and ABTS scavenging activities observed for TSM could be explained because, in addition to protein fraction, in the TSM sample, there are different components, such as phytosterols and bioactive compounds from tomato seed oil, which act as proton-donating substances. Then, these components promote greater antioxidant activity by a synergic effect [4,7]. Moreover, several studies reported that phenolic compounds showed antioxidant activity [5], and in this sense the antioxidant activity shown by TSM also could be influenced by phenolic compounds (53.38 mg Eq. GA/100g sample), which is similar to phenolic content (73.8 mg Eq. GA/100g sample) reported for seed from tomato var. saladette [31]. In contrast, in TSMD, as a result of the defatting process, the phenolic content could be reduced, as reported by Sarkar and Kaul [32], which could reinforce that antioxidant activity is primarily prompted by protein content into TSM and TSMD samples.

Metal species as ferrous ion (Fe2+) can promote the production of reactive oxygen species, which can damage living systems. In this sense, metal chelating agents may inhibit reactive oxygen species production and prevent damage to DNA, protein, and lipids into cells [33]. TSM and TSMD showed a lower chelating activity (1.1–2.2%) which was improved by *Lactobacillus* sp. fermentation, generating TSMDF with greater chelating activity (41.2%). Meanwhile, Gunyakti and Asan-Ozusaglam [34] reported DPPH scavenging activity (39.2%) and iron chelating activity (15.6%) for supernatants of *Lactobacillus* gasseri. Chi and Cho [35] reported the iron ion chelating activity of soybean meal fermented by *Lactobacillus* acidophilus as 30.53%.

The improvement of scavenging activity and iron chelating activity in TSMDF could be the result of polysaccharides hydrolysis mediated by enzymes during fermentation, and likewise the breakdown of proteins (Figure 3), which allows for the synthesis of different compounds as peptides or free amino acids which can act as chelating agents [36]. In this regard, it has been reported that there is metal chelating potential for amino acids such as methionine, glutamine, glutamic acid, lysine, or arginine within peptides sequences. In addition, other reports indicated that lactic acid bacteria during fermentation promote the synthesis of peptides with antioxidant activity and strong radical scavenging activity [37], which can explain the scavenging activity improved for TSMDF in this study.

Lactic acid bacteria have also been employed in different food sources as seeds to produce angiotensin converting enzyme inhibitor (ACEI) peptides. These peptides are the result of different conditions, such as proteases or strain used, duration of fermentation, pH, and others [38,39]. TSMDF showed almost ten-fold higher ACEI capacity than TSMD. The IC_50_ value (73.6 µg/mL) of TSMDF at 48 h after fermentation was higher than IC_50_ 180 µg/mL reported for the liquid fermentation of lentils using *Lactobacillus* plantarum. In this study, the higher ACEI was reached at 48–96 h of fermentation [38].

Interestingly, in silico analysis showed that hydrolysis fermentation could generate DPP-IV inhibitor peptides as in other vegetable sources, such as wheat gluten, lupin seeds, and fermented soybean [40]. The antihypertensive potential shown by TSMDF can be explained by ACEI peptides obtained by *Lactobacillus* sp. that, according with in silico analysis by BIOPEP, would be hydrolyzed from storage proteins of tomato seeds that showed (as previously mentioned) molecular weights between 20 and 24 kDa, 32–35 kDa, and 52–70 kDa. This agreed with the main polypeptides from tomato seed isolate reported by Mechmeche et al. [17]. This study suggests that nutraceutical properties are promoted by antioxidant peptides, ACE inhibitor peptides, DPP-IV inhibitor peptides, and chelating peptides. It is desirable to continue experimental assays to identify peptide sequences for further applications to generate nutraceutical foods or health-foods.

## 4. Materials and Methods

### 4.1. Sample Preparation

Seeds of tomato var. saladette were obtained from tomato wastes consisted mainly of seeds, peels, and pulp supplied by different industrial dining service companies from the Puebla-Tlaxcala region (19°25′44″ N, 98°9′39″W). The skins and pulp were separated from seeds by the wet method. For this, tomato wastes were immersing in distilled water 1:10 ratio (wastes:water) and mixed for 0.5 min at 66 rpm in a roller mixer at room temperature. Then, water was drained using a 100 sieve (150 µm) to drain water. This process was repeated five times reusing drained water. Finally, tomato seeds were dried in oven at 40 °C for 12 h. Seeds separation efficiency (**η**sep) was determined as:

$$\eta s\mathrm{ep}=\frac{\mathrm{Wrec}}{\mathrm{Win}}*100$$

where Wrec was the weight of seeds recovered, Win was the weight of sample (seeds, peels, and pulp) before the separation process.

Tomato seed meal (TSM) was obtained by milling the dried seeds with a blade mill. Tomato seed meal defatted (TSMD) was obtained by Soxhlet extraction using hexane at 55 °C for 6 h at a ratio 1:10 TSM:hexane (*w*/*v*). Then, TSMD was recovered and hexane residues evaporated at 40 °C under vacuum (Buchi^®^ RE 121 Rotavapor W/461 water bath laboratory).

### 4.2. Proximal Composition, Phenolic Compounds, and Proteins

The proximal composition (fiber, ash, moisture, lipids, and protein content) was determined for TSM using the methods described by AOAC: moisture, 934.06 method; ash, 930.05 method; total fat by the Soxhlet, 903.09 method; protein by the Kjeldahl using a protein conversion factor of 6.25, 978.04 method [41]. Carbohydrate content was determined by the difference.

Total phenolic compounds were determined by Follin–Ciocalteau method. Briefly, a 10-μL sample was reacted with Follin’s reagent and Na_2_CO_3_ (sodium carbonate) and incubated for two hours. After incubation, absorbance was read at 750 nm (Epoch™ Microplate Spectrophotometer, Biotek), following the method reported by [42]. Absorbance readings were compared with a gallic acid calibration curve (mg/mL) (r^2^ = 0.9909). Results were expressed as mg equivalents of gallic acid/100 g sample (mg Eq. GA).

Soluble protein concentration was determined by the Bradford method with bovine serum albumin as a standard protein (mg/mL) (r^2^ = 0.984) [43]. SDS-PAGE polyacrylamide (16%) gel technique was used to analyze protein electrophoretic profile and degraded proteins by hydrolysis during fermentation [44]. For protein extraction, 40mg TSM were added to 200uL of buffer 0.065 M Tris-HCl, 2% SDS pH 6.8, and mixed in a vortex for 1 min.

### 4.3. Techno-Functional Properties

#### 4.3.1. Water Holding Capacity (WHC)

Water holding capacity of TSM or TSMD was measured at different pH values in citrate-phosphate buffer (pH 3, 4.5, 5, 6, and 7) and tris-HCl buffer (pH 8 and 9), according to the technique reported by [45] with slight modifications. Then, one g of sample (TSM or TSMD) were mixed and stirred with 10 mL buffer during 30 min at room temperature. The slurry was centrifuged at 5000× *g* for 10 min, and the supernatant and sediment were recovered. WHC of TSM and TSMD was calculated according to:

$$\mathrm{WHC}=\frac{\left(\mathrm{Ws}-\mathrm{Wds}\right)}{\mathrm{Wi}}$$

where Ws was the weight of tube with TSM or TSMD sediment, Wds was the weight of tube with dry TSM or TSMD, and Wi was the weight of the dry whole TSM or TSMD samples.

#### 4.3.2. Oil Holding Capacity (OHC)

The oil holding capacity of TSM or TSMD was measured at different pH values, 5.0, 5.5, and 6.0 using soybean oil. Then, 1 g of sample (TSM or TSMD) was mixed and stirred with 10 mL soybean oil for 30 min at room temperature. The slurry was centrifuged at 5000× *g* for 10 min, and the supernatant and sediment were recovered. OHC of TSM and TSMD was calculated according to:

$$O\mathrm{HC}=\frac{\mathrm{Ws}-\mathrm{WDS}}{\mathrm{Wi}}$$

where Ws was the weight of tube with TSM or TSMD sediment, Wds was the weight of tube with dry TSM or TSMD, and Wi was the weight of the dry whole TSM or TSMD samples.

#### 4.3.3. Protein Solubility (PS)

To determine the solubility of proteins from TSM and TSMD, 0.1 g of meal was mixed with 1 mL citrate-phosphate buffer (pH 3, 4.5, 5, 6, 7) and Tris-HCl buffer (pH 8 and 9). The slurries were stirred at 33 rpm during 30 min in a roller mixer at room temperature. After, the slurries were centrifuged at 5000× *g* during 10 min at room temperature. The protein content in the supernatants was determined by the Bradford method. Samples of each supernatant were analyzed by SDS-PAGE. The protein solubility (PS) was determined according to:

$$\mathrm{PS}\%=\frac{\mathrm{PCs}}{\mathrm{PCm}}*100$$

where PCs was the protein content in supernatants, and PCm was the total protein content into tomato seed meal.

#### 4.3.4. Foaming Properties

One gram of TSM or TSMD was added to citrate-phosphate and Tris-HCL buffers at different pH values, and the mixture was bubbled for two minutes using an air pump (47L/h). Initial and final foam and foam coalescence were measured as proposed by [46]. The foam activity (FA) of both samples was determined according to:

$$\mathrm{FA}\%=\frac{\mathrm{Vab}-\mathrm{Vbb}}{\mathrm{Vbb}}*100$$

where Vab was the volume of samples after bubbling, and Vbb was the volume of samples before bubbling. The foam stability (Fst) was determined by half-life foam, namely time (min) necessary to reduce 50% foam volume according to reported by [11,14].

#### 4.3.5. Emulsifying Properties

One gram of TSM or TSMD was added to citrate–phosphate and Tris-HCL buffer solutions at different pH values in a 1:10 ratio (sample:buffer) and mixed for 30 min at room temperature. After, soybean oil was added in a 40:60 ratio (slurry:oil), and the mixture was stirred for two minutes using a blade mixer until an emulsion was obtained. The volume of the third phase was measured, and the emulsifying capacity (EC) for both samples was obtained according to:

$$\mathrm{EC}\%=\frac{\mathrm{Vas}}{\mathrm{Vbs}}*100$$

where Vas was the volume of the emulsion layer after stirring, and Vbs was the volume of total liquid. The emulsion stability (Est) was evaluated recording Vas during 60 min after emulsion formation at room temperature.

### 4.4. Nutraceutical Properties

Nutraceutical properties were measured to TMS and TSMD.

#### 4.4.1. Inhibitory Activity of the 2,2-diphenyl-1-picrylhydrazyl Radical Cation (DPPH)

The free radical scavenging ability of TSM alcoholic extract (1:10 flour:ethanol 70%), TSMD alcoholic extract, and TSMDF supernatants was assessed using 2,2-diphenyl-1-picrylhydrazyl radical cation (DPPH) according to the method reported by [5] with slight modifications. Briefly, 20 µL of sample adjusted at 0.1 mg/mL (of protein content) with ethanol were added to 200 µL of a 150 µmol DPPH solution, and the mixture was left to rest for 30 min. Then, the absorbance at 517 nm was recorded, against 70% ethanol as a blank. Results were reported as μM ET (trolox equivalent) and %I (percent inhibition), according to the following equation:

$$\%I=1-\left(\frac{\mathrm{As}}{\mathrm{Ac}}\right)*100$$

where As was the absorbance of sample, and Ac was the absorbance of the control which contained 20 uL ethanol instead samples.

#### 4.4.2. Inhibitory Activity of the Radical Cation of 2,2′azinobis-(3-ethylbenzothialin)-6-sulfonic acid (ABTS)

The scavenging of 2,2′azinobis-(3-ethylbenzothialin)-6-sulfonic acid (ABTS) was carried out as reported by [17] with some modifications. ABTS^+^ was prepared in 2 mM destilled water with 2.45 mM potassium persulfate. The working solution was incubated during 12 h in the dark at room temperature. Then, the working solution was diluted with 0.1 M phosphates buffer (pH 7.4) to obtain an absorbance value to 0.70 ± 0.02 at 734 nm. To measure the antioxidant capacity, 10 μL of TSM aqueous extract, TSMD aqueous extract, and TSMDF supernatants (adjusted to 1.1 mg/mL of protein content) was added to 200 µL of the ABTS working solution diluted and allowed to react for 6 min after mixing. Subsequently, the absorbance was read at 734 nm. Results were reported as μM ET (trolox equivalent) and %I (percent inhibition) according to the following equation:

$$\%I=1-\left(\frac{\mathrm{As}}{\mathrm{Ac}}\right)*100$$

where As was the absorbance of sample, and Ac was the absorbance of the control which contained 10 uL water samples instead.

#### 4.4.3. Iron Chelating Activity

To evaluate the iron chelating activity (ICA) 50 µL of FeSO_4_ (2 mM) was added to 1 mL of TSM aqueous extract, TSMD aqueous extract, and TSMDF supernatants. The samples were stirred and incubated by 10 min at room temperature. Then 100 µL of ferrozine (5 mM) were added and, after 10 min, the absorbance at 562 nm was read [47]. A blank was prepared using water instead of ferrozine; the results were expressed as % (percent quelation). The following equation was used to determine ICA (%):

$$\%I\mathrm{CA}=1-\left(\frac{\mathrm{As}}{\mathrm{Ac}}\right)*100$$

where As was the absorbance of sample and Ac was the absorbance of the control which contained water instead of ferrozine.

#### 4.4.4. Angiotensin Converting Enzyme Inhibition (ACEI)

Supernatants obtained at 0, 12, 24, 36, 48, and 60 h of fermentation were used for measuring the Angiotensin Converting Enzyme inhibitory activity (ACEI) according to reported by [29]. All assays were carried out in triplicate. The ACEI was reported as IC_50_ value, namely the concentration of hydrolyzed protein that inhibit 50% of the ACE activity.

### 4.5. Enhancement of Nutraceutical Properties by Fermentation

*Lactobacillus* sp. strain was obtained from CIBA-IPN collection of microorganisms. Pre-cultures were grown overnight in MRS broth at 37 °C. Subsequently, cells were harvested by centrifugation at 10,000× *g* for 10 min at 10 °C. The cell pellet was washed with sterile isotonic solution and resuspended in sterile isotonic solution to prepare bacterial suspension.

TSMD 1:5 ratio (sample:distilled water) was used to prepare a fermentation broth supplemented with sucrose (1.1%). Shake flask fermentations were performed in Erlenmeyer flasks containing 30 mL of broth inoculated with 1.3 × 10^4^ CFU (Colony Forming Units). Cultures were incubated at 37 °C for 60 h. Samples were taking at 0, 12, 24, 36, 48, and 60 h of fermentation; were harvested by centrifuging at 10,000× *g* for 10 min at 10 °C. The supernatants were decanted; cell pellets and supernatants were stored at −20 °C for further analysis. The total protein content in all samples harvested was measured by the Bradford method, as well as the carbohydrate content by the phenol-sulfuric method in microplate [48]. Samples of each stage of fermentation were analyzed by SDS-PAGE.

### 4.6. In Silico Analysis: Biopeptides

BIOPEP database was used to evaluate the potential of TSMD as bioactive peptide source through hydrolysis of proteins by proteases from *Lactobacillus* spp. [49]. For this, Enzyme(s) Action tool (BIOPEP) was used to carry out proteolysis selecting prolyl oligopeptidase (EC 3.4.21.26), Xaa-Pro dipeptidase (EC 3.4.13.9), and proteinase P1 (EC 3.4.21.96) which are enzymes implicated in *Lactobacillus* spp. metabolism confirmed by the BRENDA database [50]. Four protein sequences from tomato seed were selected from UniProtKB database (http://www.uniprot.org/) with entries: A0A3Q7IWI5 (W15, seed storage protein), A0A3Q7I2A5 (A5, seed storage protein), A0A3Q7I4W4 (W4, seed storage protein), and K4CWS6 (S6, glucocyltransferase), plus two protein sequences from RCSB PDB database (http://www.rcsb.org/) with accession numbers 6L4M (vicilin) and 4I8Q (aminoaldehyde dehydrogenase). All of them are proteins involved in seed development or seed germination of tomato.

After in silico digestion, all data collected were analyzed and frequency activity (fac) was determined according to the equation:

$$\mathrm{fac}=\frac{f}{F}*100$$

where f is the number of fragments with a certain bioactivity and F is the number of total fragments with different bioactivities.

### 4.7. Statistical Analysis

The SAS 7.0 statistic program (SAS Institute Inc., Cary, North Carolina) was used to analyze the data collected from experimental assays. All assays were done in triplicate and results are presented as mean ± standard deviation (SD). ANOVA analysis and a Tukey test were carried out to observe significant differences (*p* < 0.05).

## 5. Conclusions

The techno-functional properties for TSM and TSMD are higher at alkaline conditions (pH 8–9). In comparison with TSM, TSMD showed higher water holding capacity (WHC ≈ 32%), higher oil holding capacity (OHC ≈ 13%), higher protein solubility (49–58%), more than 10 times foaming activity (FA), more than 50 times foam stability (Fst), as well as improved emulsifying activity (EA) and emulsion stability (Est), which were better at pH 9. After 48 h of fermentation, TSMDF doubled its antioxidant activity, a significant increase in its iron-chelating activity was also observed, and angiotensin-converting enzyme inhibition (ACEI) was more than 10 times higher than TSMD. Therefore, the strategy proposed in this study could be an option for exploitation of tomato wastes.

## Figures and Tables

**Table 1:** Proximal composition of TSM sample.

Component	(%)
Moisture	9.93 ± 0.73
Ash	3.92 ± 0.11
Proteins	26.93 ± 0.12
Lipids	14.02 ± 1.12
Carbohydrates *	18.21
Fiber	26.99 ± 2.92

Results are mean of triplicate assays, ± standard deviation. * Result obtained by difference.

**Table 2:** Techno-functional properties of tomato seed meal (TSM) and tomato seed meal defatted (TSMD).

Techno-Functional Property	TSM				TSMD			
	pH 6	pH 7	pH 8	pH 9	pH 6	pH 7	pH 8	pH 9
WHC *	3.18 ± 0.11 ^a^	3.25 ± 0.25 ^a^	3.27 ± 0.11 ^a^	3.26 ± 0.05 ^a^	4.2 ± 0.2 ^b^	4.22 ± 0.23 ^b^	4.27 ± 0.17 ^b^	4.31 ± 0.31 ^b^
OHC ^+^	2 ± 0.1 ^a^	-	-	-	2.3 ± 0.15 ^b^	-	-	-
Protein solubility (%)	41.6 ± 1.7 ^a^	45.7 ± 3.2 ^a^	50.1 ± 0.2 ^b^	55.9 ± 1.1 ^c^	64.2 ± 1.1 ^d^	70.5 ± 0.7 ^e^	79.4 ± 2.7 ^f^	83.7 ± 1.21 ^f^
FA (%) ^«^	<2.5 ^d^	<2.5 ^d^	<2.5 ^d^	<2.5 ^d^	16.8 ± 0.3 ^a^	17.01 ± 0.04 ^a^	26.3 ± 0.13 ^b^	32.1 ± 0.2 ^c^
Fst (min) ^^^	<0.5 ^e^	<0.5 ^e^	<0.5 ^e^	<0.5 ^e^	1.7 ± 0.2 ^a^	2.1 ± 0.01 ^b^	29.3 ± 0.5 ^c^	38.1 ± 0.1 ^d^
EA (%) ^α^	<4 ^h^	20 ± 0.01 ^a^	87.5 ± 0.06 ^b^	33.3 ± 0.02 ^c^	6.7 ± 0.02 ^d^	5.0 ± 0.03 ^e^	83.3 ± 0.07 ^f^	60.5 ± 0.06 ^g^
Est (%) ^β^	<1 ^h^	68.2 ± 0.01 ^a^	68.5 ± 0.04 ^b^	62.0 ± 0.05 ^c^	67.3 ± 0.09 ^d^	71.7 ± 1.1 ^e^	60.1 ± 0.07 ^f^	69.8 ± 0.1 ^g^

* WHC, water holding capacity (g water/g sample); ^+^ OHC, oil holding capacity (g oil/g sample); ^«^ FA, foam activity; ^^^ Fst, foam stability as time (min) to collapse 50% of foam; ^α^ EA, emulsifying activity; ^β^ Est, Emulsion stability. Different superscripts letters (a–h) (by rows) indicate significant difference (*p* < 0.05).

**Table 3:** Nutraceutical properties of tomato seed meal (TSM), tomato seed meal defatted (TSMD), and tomato seed meal defatted and fermented (TSMDF).

Sample	DPPH		ABTS		Iron Chelating Activity	ACEI
	μM Eq Trolox/100 g Sample	*Scavenging Activity* (% I)	μM Eq Trolox/100 g Sample	*Scavenging Activity* (% I)	(%)	Inhibition %
TSM	375.6 ± 4.6 ^a^	41.5 ± 2.3 ^a^	187.8 ± 2.2 ^a^	20.2 ± 0.3 ^a^	1.1 ± 0.3 ^a^	4.41 ± 1.3 ^a^
TSMD	355.1 ± 4.8 ^b^	38.9 ± 5.3 ^a^	177.3 ± 1.8 ^b^	19.3 ± 0.05 ^b^	2.2 ± 0.01 ^b^	7.9 ± 0.1 ^b^
TSMDF (48 h)	792.5 ± 5.3 ^c^	86.8 ± 1.3 ^b^	419.1 ± 6.5 ^c^	45.6 ± 0.2 ^c^	41.2 ± 1.1 ^c^	83.7 ± 0.7 ^c^

Different superscripts letters a–c (by columns) indicate significant difference (*p* < 0.05).

## Supplementary Materials

The following are available online at https://www.mdpi.com/1420-3049/25/18/4235/s1, Figure S1: Holding capacity of fluids, Figure S2: Protein solubility of tomato seed meal, Figure S3: Emulsifying activity of tomato seed meal.
