# Supplementary material for: Assessment of Techno-Functional and Nutraceutical Potential of Tomato (Solanum lycopersicum) Seed Meal
Source: Molecules. 2020 Sep 15;25(18):4235. doi: 10.3390/molecules25184235 (PMC7571165; doi:10.3390/molecules25184235)
Supplement: Supplementary file 1 [file molecules-25-04235-s001.pdf]

Supplementary material

Assessment of techno-functional and bioactive potential of tomato *(Solanum lycopersicum)* seed meal

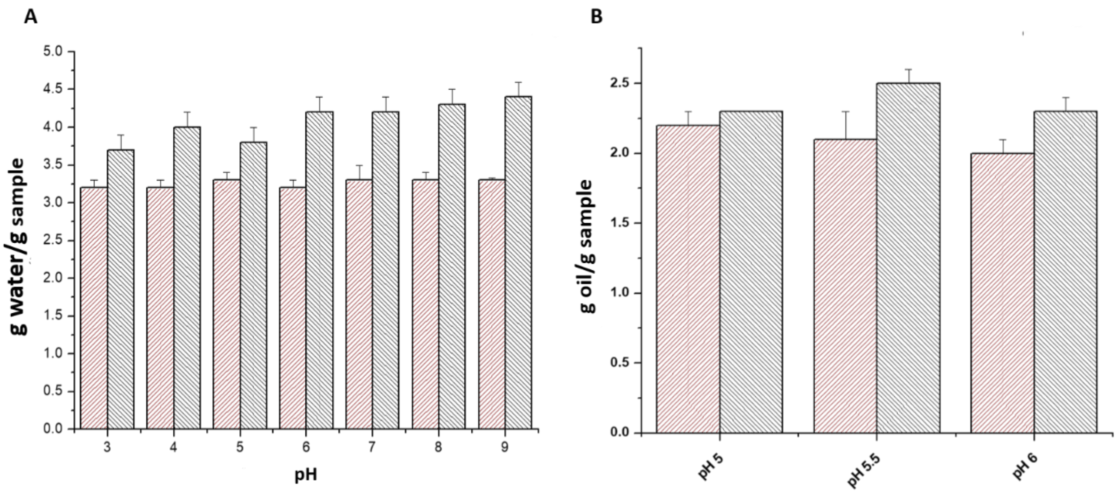

Fig. S1. Holding capacity of fluids. A, Water Holding Capacity; B, Oil Holding Capacity. Red bars are corresponding to TSM sample; gray bars are corresponding to TSMD sample.

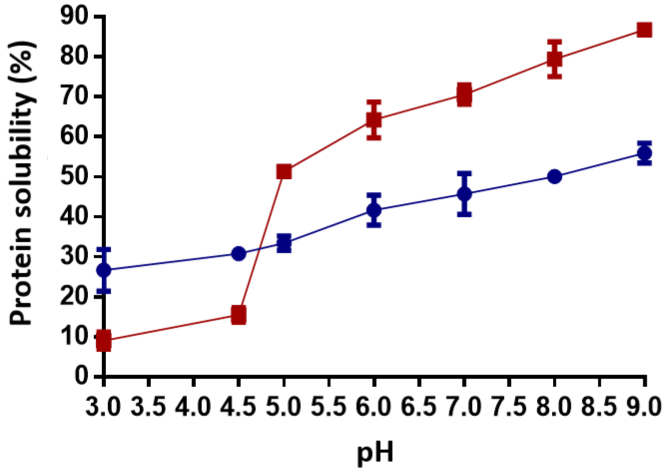

Fig. S2. Protein solubility of tomato seed meal. Blue line, shows protein solubility for TSM sample; red line, shows proteins solubility for TSMD sample.

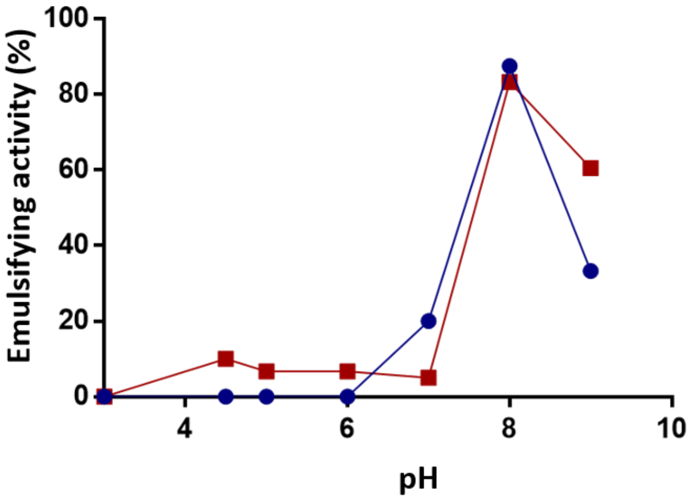

Fig. S3. Emulsifying activity of tomato seed meal. Blue line, shows protein solubility for TSM sample; red line, shows proteins solubility for TSMD sample.
